# Sex-Related Differences in Clinical Outcomes in Patients with Atrial Fibrillation and Coronary Artery Disease: A Sub-Study of the MISOAC-AF Randomized Controlled Trial

**DOI:** 10.3390/jcm11195843

**Published:** 2022-10-01

**Authors:** Alexandra Bekiaridou, Athanasios Samaras, Anastasios Kartas, Andreas S. Papazoglou, Dimitrios V. Moysidis, Vasiliki Patsiou, Stefanos Zafeiropoulos, Antonios Ziakas, George Giannakoulas, Apostolos Tzikas

**Affiliations:** 1 First Department of Cardiology, School of Medicine, Faculty of Health Sciences, AHEPA University Hospital, Aristotle University of Thessaloniki, 54636 Thessaloniki, Greece; 2 Institute of Bioelectronic Medicine, Feinstein Institutes for Medical Research, Manhasset, NY 11030, USA; 3 Interbalkan European Medical Center, Asklipiou 10, Pylaia, 55535 Thessaloniki, Greece

**Keywords:** atrial fibrillation, coronary artery disease, sex, prognosis

## Abstract

Background: There is limited “real-world” data on the prognostic role of gender in comorbid atrial fibrillation (AF) and coronary artery disease (CAD). Methods: In this post-hoc analysis of the MISOAC-AF randomized trial (NCT: 02941978), consecutive patients with AF and CAD who were discharged from the cardiology ward between 2015 and 2018 were included. Multivariable Cox-regression analysis was performed for all-cause mortality and cardiovascular (CV) mortality. Competing-risk analysis was performed for the outcomes of stroke or systemic embolism, major bleeding, AF- or heart failure (HF)-related hospitalization, adjusted for the competing risk of all-cause death. Results: Of 1098 patients with AF, 461 patients with comorbid CAD were analyzed. Women were older and more likely to have a history of diabetes mellitus and valvular heart disease, while men were more likely to have a history of smoking or myocardial infarction. Over a median follow-up of 31 months, 143 (43.4%) men and 71 (53.7%) women died. Women were at a higher risk for all-cause mortality (adjusted hazard ration [aHR] 1.65; 95% confidence interval [CI] 1.14–2.38) and stroke or systemic embolism (aHR 3.52; 95% CI 1.46–8.49) compared to men. The risks of CV mortality, major bleeding, AF-related hospitalization, and HF-related hospitalization were similar between genders. Conclusions: In recently hospitalized patients with AF and comorbid CAD, the female gender was independently associated with increased all-cause mortality and thromboembolic events.

## 1. Introduction

Atrial fibrillation (AF) constitutes the most common sustained arrhythmia, and a progressive global burden, expected to affect 14–17 million patients in Europe by 2030 [1]. In patients with AF, coronary artery disease (CAD) coexists at a rate of 13% to 18% in large trials [2,3]. AF occurs in 5–40% of CAD patients, especially after cardiac surgery [4]. AF and CAD share common risk factors; age, smoking, sleep apnea, high blood pressure, obesity, and diabetes mellitus (DM) [5]. The frequent coexistence of AF and CAD is of prognostic significance, accounting for increased morbidity, complications, recurrent hospitalizations, and healthcare costs [5]. Additionally, AF and CAD substantially burden the patient’s management regarding the appropriate, guideline-guided administration of anticoagulants with antiplatelets and the duration of therapy [5].

Lately, there has been a rising interest in gender-specific patient profiling. This includes epidemiology and treatment of cardiovascular (CV) diseases such as AF and CAD [6]. CV diseases account for increased mortality in women, even though the notion that they primarily affect men remains [7]. Gender-specific differences in epidemiology are driven by peculiarities in gene expression, hormones, and lifestyle [7]. The incidence of AF is higher in men than women but rises steadily with advancing age and hormonal changes in menopause. Men commonly have CAD at a younger age, which in turn predisposes them to AF. On average, females grow older and have different comorbidities than males [8]. Thus, there is an increasing need to study characteristics and clinical outcomes under the scope of gender.

Against this background, we performed a post-hoc analysis on a cohort of well-characterized patients with comorbid AF and CAD. The objective of this study was to assess the influence of female gender on clinical characteristics and outcomes.

## 2. Methods

This is a post-hoc analysis of MISOAC-AF (Motivational Interviewing to Support Oral AntiCoagulation Adherence in patients with non-valvular Atrial Fibrillation, ClinicalTrials.gov identifier: NCT02941978) trial [9]. MISOAC-AF was a prospective, randomized controlled trial, conducted in a single academic hospital (AHEPA University Hospital, Greece) from December 2015 through June 2018. The trial investigated the effect of patient–physician interviews and scripted guidance in strengthening the compliance of AF patients to oral anticoagulants (OACs) [10]. 

### 2.1. Data Sources

The electronic database includes baseline demographics, medical history, medication profiles, and follow-up data of patients who were hospitalized with AF. The vital status of all patients was additionally verified through the Greek Civil Registration System and all the events were adjudicated. The study was approved by the Aristotle University of Thessaloniki review board and was conducted in compliance with the Declaration of Helsinki.

### 2.2. Study Population

The study comprised adult patients, consecutively enrolled in the prospective cohort of the MISOAC-AF trial. Comorbidity of AF with CAD was the shared feature of all patients included, irrespective of the discharge diagnoses from the Cardiology Department of AHEPA Hospital of Thessaloniki, Greece. The presence of end-stage disease not permitting the patients’ follow-up, as well as the absence of data (unavailable or unknown) concerning the presence of CAD, were the exclusion criteria for the present study.

### 2.3. Study Variables

Data concerning gender, clinical profiles, comorbidities, discharge diagnoses and medication, fatal and non-fatal CV events during follow-up were extracted and analyzed. The CHA_2_DS_2_-VASc and HAS-BLED scores were calculated using available data [11]. 

### 2.4. Study Outcomes

We assessed outcomes according to gender (males versus females). The assessed outcomes were all-cause mortality, i.e., death from any cause, stroke or systemic embolism, major bleeding, CV death, and hospitalizations related to AF or HF. 

### 2.5. Definition of Covariates

All data collection was based on the standard definitions, as defined in the study [12,13]. AF was defined as previously recorded in medical history or new-onset AF occurring during hospitalization. The latter concerned irregular heart rhythm for more than 30 s, without detectable P waves, captured by a 12-lead electrocardiogram or a 24-h Holter monitor [11]. CAD was defined as history of any of the following: (i) imaging modality demonstrating ≥ 50% coronary artery stenosis, (ii) previous CABG/PCI, (iii) previous MI. Patients were considered to have valvular heart disease if they had (i) a history of at least moderate valvular heart disease, (ii) baseline echocardiographic evidence of at least moderate valvular heart disease, or (iii) a history of previous valve surgery reported on the trial case report form. Stroke was defined as a permanent, focal, neurological deficit confirmed by imaging modalities. Transient ischemic attack was defined as new neurologic symptoms or a deficit lasting less than 24 h with no new infarction on neuroimaging (if available). Systemic embolism was defined as signs or symptoms of peripheral ischemia supported by evidence of embolism from surgical specimens, autopsy, angiography, vascular imaging, or other objective testing. Major bleedings were intracranial hemorrhages and upper and lower gastrointestinal bleedings. CV death was defined as a death related to cardiac causes or stroke.

### 2.6. Statistical Analysis

Continuous variables were reported as means with standard deviations (SD) or medians with interquartile range (IQR), and categorical variables as frequencies (%). Between-group comparisons were conducted using the Pearson chi-square or Fisher’s exact test for categorical variables and the Wilcoxon rank-sum or Student’s t-test test for continuous variables, depending on the normality of data distributions. Cumulative event rates were estimated by Kaplan–Meier estimation. Test covariates included: age, body mass index (BMI), gender, prior stroke, CAD or prior coronary revascularization procedure, renal function, AF subtype (first-diagnosed, paroxysmal, persistent, or permanent), use of OAC, angiotensin converting enzyme inhibitors or angiotensin II receptor blockers (ACEI-ARB), and rate control medication after discharge. To calculate the risk of the secondary outcomes (CV death, stroke or systemic embolism, major bleeding, AF- and HF- hospitalizations) during follow-up, a modified Cox regression analysis was utilized, accounting for the competing risk of all-cause death [14]. Subgroup analysis was also performed to explore patient characteristics, including HF, DM, type of AF, type of OAC used, age, LVEF, and renal function. Missing data in the multivariable models were replaced by means of multiple imputations, using chained equations. A *p* value < 0.05 was considered statistically significant. 

Data management and statistical analysis were performed using SPSS version 27 (SPSS Inc., Chicago, IL, USA) software and Stata v15.1 (StataCorp, College Station, TX, USA) packages.

## 3. Results

### 3.1. Baseline Characteristics, Demographics, and Medical History 

A total of 1140 patients were screened for the MISOAC-AF trial, out of which 1063 patients were eligible and underwent randomization. After randomization, 54 patients did not receive OAC treatment and, thus, were excluded from the initial study. Among 1009 patients that were enrolled in the trial, 461 (41.9%) had AF and CAD (mean age 74.7 ± 9.0 years) and were included in this post-hoc analysis. Males with CAD made up 71.3% (N = 329) of the study population and females with CAD made up 28.7% (N = 132). 

Baseline characteristics, demographics, medical history, and medication at the discharge of patients with AF and CAD grouped by gender are described in Table 1. Female patients were older compared with men (mean age 77.2 vs. 73.7; *p* < 0.001). Men were more likely to be smokers and have a history of CABG or MI (all *p* < 0.001). Furthermore, they were more likely to have a pacemaker or implantable cardioverter/defibrillator (14.7% vs. 6.1%; *p* = 0.004). Overall, women were more likely to have DM (55% vs. 37.9%; *p* = 0.001), VHD (72.3% vs. 56.4%; *p* = 0.003) and a higher CHA_2_DS_2_-VASC score (6.0 ± 1.4; *p* < 0.001). Treatment at discharge did not reveal significant differences among genders.

### 3.2. Outcomes According to Gender

Over a median follow-up period of 32 months, the outcome of all-cause death occurred in 143 of 329 men and 71 of 132 women (43.4% vs. 53.7%, annual rates 16.3% vs. 20.3%). After adjusting for covariates, female sex was independently associated with all-cause mortality (adjusted HR [aHR] 1.65; 95% confidence interval [CI] 1.14–2.38; *p* = 0.008). 

**Figure 1:**
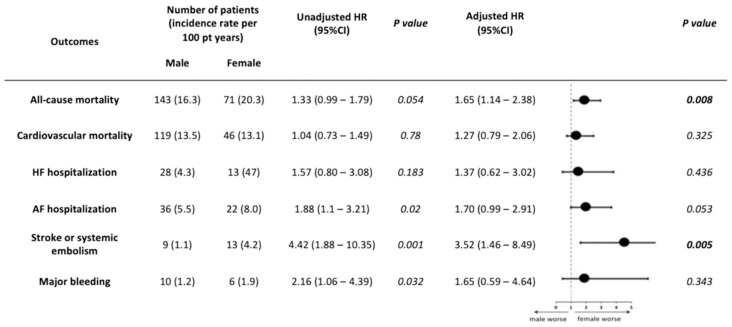
Risk of clinical outcomes according to gender. Abbreviations: AF, atrial fibrillation; CI, confidence interval; HF, heart failure; HR, hazard ratio; pt, patient.

**Figure 2:**
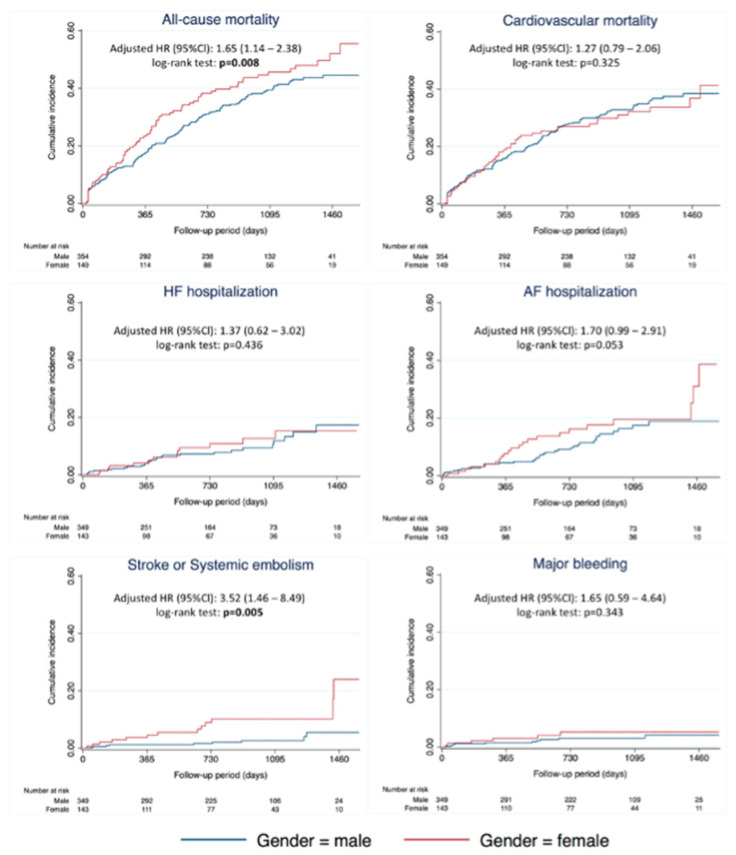
Cumulative incidence of clinical outcomes according to gender. Abbreviations: AF, atrial fibrillation; CI, confidence interval; HF, heart failure; HR, hazard ratio.

Regarding CV mortality (aHR 1.27; 95% CI 0.79–2.06; *p* = 0.325), AF hospitalization (aHR 1.7; 95% CI 0.99–2.91; *p* = 0.053), HF hospitalization (aHR 1.37; 95% CI 0.62–3.02), and major bleeding (aHR 1.65; 95% CI 0.59–4.64), they did not demonstrate any significant differences between groups (Figure 1 and Figure 2). Female gender was associated with stroke or systematic embolic events (aHR 3.52; 95% CI 1.46–8.49; *p* = 0.005), with an annual rate of 1.1% for men vs. 4.2% for women.

### 3.3. Subgroup Analysis

**Figure 3:**
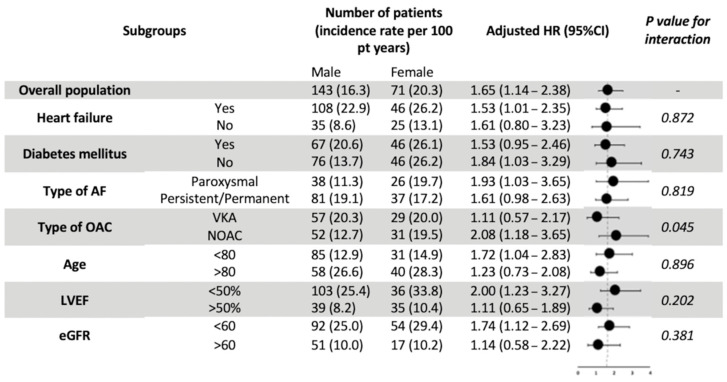
Major subgroup analyses of all-cause mortality by gender. Abbreviations: AF, atrial fibrillation; eGFR, estimated Glomerular Filtration Rate; LVEF, left ventricular ejection fraction; OAC, oral anticoagulants; VKA, Vitamin-K antagonists; NOAC, Non-Vitamin K antagonist oral anticoagulants; HR, hazard ratio.

Subgroup analyses for all-cause death did not demonstrate any statistical significance between subgroups of HF, DM, AF pattern, age, LVEF, or renal function (Figure 3). However, the female gender appeared to carry significantly more prognostic value for all-cause mortality in NOAC users compared with VKA users (aHR 2.08 vs. 1.11; *p* for interaction = 0.045). 

## 4. Discussion

This study explored the influence of female gender on the overlapping entities of AF and CAD, based on a contemporary population of a randomized trial. The main findings concerning the AF and CAD comorbidity were: (i) female gender was associated with 65% increase on all-cause mortality, (ii) risk for stroke or embolic events was more than two times higher in females than that in males. All outcomes were adjusted for known confounders; non-fatal outcomes were additionally adjusted for the competing risk of all-cause mortality.

Our study confirms the results from previous studies and adds substantial data on the gender-specific epidemiology of AF and CAD. In the in-hospital setting, we observed that women were older and more likely to have a history of DM and VHD compared with their male counterparts. DM is a significant modifiable risk factor for CAD and aggravates the risk for AF [7,14,15]. Women with DM are reported to be 24% more likely to develop AF than men with DM and have a 3-fold higher risk of fatal CAD compared with non-diabetic women [7,16]. Diabetic women may also not have symptoms; thus, the presence of DM constitutes a significant burden on women prognosis [17]. Interestingly, women with CAD and AF also showed a significant history of VHD compared with men. The suggested pathophysiological mechanism is an age-dependent atherosclerotic process, which is a common etiology of aortic stenosis; the most prevalent valvular lesion among CAD patients [18,19]. 

The interplay between risk factors, comorbidities, and the development of AF and CAD seems to directly affect all-cause mortality in females. Poorer outcomes of women with ischemic heart disease are reported in the bibliography but have yet to be elucidated [6]. Several other reports demonstrate higher mortality rates among women [20,21]. Νon-obstructive CAD, microvascular ischemic disease, and MI with non-obstructive coronary arteries (MINOCA) are more prevalent in women. At the same time, men are more likely to have obstructive CAD and experience MI [22]. The various pathophysiological pathways and disease manifestations are mirrored in females’ often atypical clinical presentation. As a result, women may be subjected to inappropriate treatment, which might explain the discrepancies in mortality rates [21]. 

An interesting, yet unexpected, finding of our study was the observed higher prognostic value of gender in NOAC users. This could not be explained by a potential off-labeled NOAC underdosing in women, since the proportions of underdosed NOAC users did not differ between genders (25.3% in women vs. 22.1% in men, *p* = 0.585). Adherence to OACs using a proportion of claimed prescriptions during the follow-up period, which corresponded to the proportion of days covered (PDC) by an OAC agent, also could not explain the observed prognostic discrepancy. Of note, the mean PDC between men and women with CAD did not differ among VKA (0.69 vs. 0.65, *p* = 0.243) or among NOAC users (0.83 vs. 0.86, *p* = 0.647). Hence, the observed difference in prognosis between genders in NOAC users could not be explained in our study, and warrants investigation in future studies.

Our analysis depicted the higher risk of stroke and embolism in women, and the results aligned with previous research [23]. Regarding therapy, our results were compatible with the global anticoagulant registry in the FIELD (GARFIELD)-AF registry, which showed no significant difference in the overall rate of anticoagulant use between women and men [24]. In previous registries, inequalities in anticoagulant treatment were more evident. Women were more often treated with aspirin and less often with OACs [25]. Lately, the role of female gender in the CHA_2_DS_2_-VASc score and the decision for initiation of anticoagulation therapy has been debated [26]. Available research leans towards the opinion that female gender is not a risk factor but rather a risk modifier. Females with zero non-gender-related risk factors for stroke (CHA_2_DS_2_-VASc score equal to (1) appear to have a similar thromboembolic risk to men, with a score of 0 [27].

## 5. Strengths and Limitations

This study, being single-centered, has the advantage of homogeneity in patient care in one cardiology ward. Moreover, this registry provided the analysis with real-world data. Regarding limitations, the current study suffers from limitations pertinent to sub-group analysis of randomized controlled trials, where the strata were not prespecified in the original study. The medium-sized sample resulted in smaller subgroups; therefore, interpretation should be considered in this context. 

## 6. Conclusions

In patients with AF and CAD who were hospitalized in the cardiology ward, females had a higher comorbidity burden at baseline than males. Female gender was an independent predictor for all-cause mortality and thromboembolic events. Careful interpretation of the results in everyday clinical practice and further research on larger cohorts are warranted.

## Figures and Tables

**Table 1:** Baseline characteristics stratified by gender.

	Women with CAD *(n = 132)*	Men with CAD *(n = 329)*	*p*-Value
**Characteristics, (mean ± SD)**			
Age (years)	77.21 ± 8.9	73.76 ± 8.9	**<0.001**
Body mass index (kg/m^2^)	29 ± 4.7	28.4 ± 5.1	0.26
Glomerular filtration rate by CKD-EPI (mL/min/1.73 m^2^)	55.53 ± 27.8	66.03 ± 32.5	**0.006**
High sensitivity Troponin-T (pg/mL)	190.7 ± 943	160.7 ± 714.9	0.96
NT-pro BNP (pg/mL)	5222.8 ± 6546.4	5155.8 ± 8004.8	0.95
LVEF	49.92 ± 11.1	43.54 ± 12.9	**<0.001**
Length of Hospitalization (days)	8.03 ± 8.8	8.69 ± 8	0.45
Reason for hospitalization			
Atrial fibrillation	43 (32.6)	117 (35.6)	0.85
Congestive heart failure	16 (12.1)	32 (9.7)	0.78
Acute coronary syndrome	11 (8.3)	78 (23.7)	0.34
Valvular heart disease	32 (24.2)	15 (4.6)	0.21
Other cardiovascular reason	30 (22.7)	87 (26.4)	0.72
**Clinical history, No (%)**			
**Current smokers**	40 (30.5)	239 (73.1)	**<0.001**
Dyslipidemia	91 (69.5)	211 (64.5)	0.31
Systolic blood pressure (mean ± SD, mmHg)	141.1 ± 26.9	141.5 ± 25.6	0.86
**Diastolic blood pressure (mean ± SD, mmHg)**	76.8 ± 16.5	81.61 ± 14	**0.005**
Hypertension	112 (85.5)	274 (83.8)	0.65
AF duration (mean ± SD, days)	2134.4 ± 2646.2	2232.1 ± 3080	0.85
Newly-diagnosed AF	45 (34)	132 (40.1)	0.31
**Type of existing AF**			
Paroxysmal	64 (48.4)	153 (46.5)	0.31
Persistent (>1 year) AF	45 (34)	102 (31)	0.16
**PCI/CABG**	67 (20.5)	260 (79.5)	**<0.001**
**Prior MI**	55 (42)	173 (52.9)	0.035
Cardiac arrest	5 (3.8)	21 (6.4)	0.27
**Pacemaker**	**6 (4.5)**	**42 (12.7)**	**<0.01**
ICD	2 (0.4)	6 (0.2)	0.053
Congenital heart disease	2 (1.5)	5 (1.5)	0.99
**Diabetes mellitus**	72 (55)	124 (37.9)	**0.001**
Vascular disease	98 (74.8)	252 (77.1)	0.6
Pulmonary disease	26 (19.8)	57 (17.4)	0.54
Ischemic stroke, TIA, undefined stroke	25 (19.2)	57 (17.5)	0.66
Heart failure	80 (61.5)	203 (62.1)	0.91
**Valvular heart disease**	86 (72.3)	162 (56.4)	**0.003**
Persistent and permanent AF	67 (51.9)	174 (54.4)	0.63
Paroxysmal AF	51 (39.5)	114 (35.6)	0.43
ACS (STEMI, NSTEMI, UA)	24 (21.1)	65 (23.8)	0.55
History of major bleeding	21 (16)	52 (15.9)	0.97
Prior cardioversion	1 (0.8)	15 (4.6)	0.22
Prior ablation	0 (0)	9 (2.8)	0.055
**Treatment No (%) and risk stratification scores (mean ± SD)**			
Use of antiplatelets at discharge			
Aspirin	16 (14.2)	33 (11.8)	0.153
Clopidogrel	9 (8)	44 (15.7)	0.153
Both	15 (13.3)	45 (16.1)	0.153
Use of oral anticoagulant(s)			
VKA	50 (43.5)	99 (34.7)	0.13
NOAC	50 (43.5)	129 (45.3)	0.13
Dabigatran	9 (7.8)	24 (8.7)	0.23
Rivaroxaban	19 (16.5)	61 (21.4)	0.23
Apixaban	22 (19.1)	44 (15.4)	0.23
**ACEi-ARB**	56 (42.4)	175 (53.2)	**0.016**
Rate control medication	106 (80.9)	257 (79.6)	0.74
B-blockers	99 (75.6)	245 (71.9)	0.83
Digoxin	1 (0.8)	1 (0.3)	0.83
B-blockers and digoxin	6 (4.6)	11 (3.4)	0.83
Rhythm control medication	25 (20.8)	73 (23.9)	0.49
Propafenone	4 (3.3)	3 (1)	0.18
Amiodarone	20 (16.7)	62 (20.3)	0.18
Sotalol	1 (0.8)	8 (2.6)	0.18
Use of statin	74 (60.2)	203 (66.8)	0.195
**CHA_2_DS_2_-VASc (mean ± SD)**	6.08 ± 1.47	4.61 ± 1.56	**<0.001**
HAS-BLED Mean (mean ± SD)	2.35 ± 1.09	2.21 ± 1.15	0.62

Values are N (%), mean (SD), DM: Diabetes mellitus, no-DM: without Diabetes mellitus, STEMI: ST-Elevation myocardial infarction, NSTEMI: non-ST segment elevation infarction, CKD-EPI: Chronic Kidney Disease Epidemiology Collaboration, CAD: Coronary artery disease, VKA: Vitamin-K, antagonists, NOAC: Novel oral anticoagulants, ICD: Implantable cardioverter defibrillator, MI: Myocardial Infarction, ACEi: angiotensin-converting enzyme inhibitor, ARB: angiotensin receptor blocker. In bold are all the statistically significant results.

## Data Availability

Data can be requested from the corresponding author.

## Abbreviations

AF	Atrial Fibrillation
ACS	Acute Coronary Syndrome
CAD	Coronary Artery Disease
CV	Cardiovascular
MINOCA	Myocardial infarction with non-obstructive coronary arteries
